# Neural Mechanisms Underlying Reading Impairment in Children Learning a Second Language: A Review

**DOI:** 10.3390/bs15101382

**Published:** 2025-10-11

**Authors:** Jia Zhang, Bingkun Li, Hehui Li

**Affiliations:** 1 School of Psychology and Cognitive Science, Beijing Language and Culture University, Beijing 100083, China; zhangjia@blcu.edu.cn; 2 Key Laboratory of Language and Cognitive Science (Ministry of Education), Beijing Language and Culture University, Beijing 100083, China; 3 National Research Center for Language and Well-Being, Shanghai 200240, China; 4 National Key Laboratory of Human Factors Engineering, China Astronaut Research and Training Center, Beijing 100094, China; 5 China Astronaut Research and Training Center, Beijing 100094, China; 6 Center for Brain Disorders and Cognitive Sciences, School of Psychology, Shenzhen University, Shenzhen 518060, China

**Keywords:** second language, reading impairment, neuroimaging, cross-linguistic, commonalities and differences

## Abstract

With the growing prevalence of bilingual education, second language (L2) reading impairment has garnered increasing attention. While extensive research has focused on reading impairment in the first language (L1), the neural mechanisms underlying L2 reading impairment have not been systematically and adequately explored. Neuroimaging studies have identified functional abnormalities in regions such as the occipitotemporal and temporoparietal cortices in children with L2 reading impairment. These neural patterns exhibit both commonalities and differences compared to those observed in L1 reading impairment. In this review, we summarized the neurocognitive characteristics of reading impairment in a L2 by comparing them with those in L1. Additionally, we proposed potential mechanisms driving these cross-linguistic differences and commonalities. Finally, we highlighted directions for future research to advance the neurocognitive understanding of L2 reading impairment.

## 1. Introduction

Reading proficiency serves as the cornerstone of individual learning and development, as well as a central educational objective during foundational schooling. However, not all learners progress smoothly to become fluent readers. The British Dyslexia Association (BDA) defines developmental dyslexia as a lifelong, genetic, neurological specific learning difficulty which primarily affects the skills involved in accurate and fluent word reading and spelling. It affects all genders, ethnicities and social groups, and its impact can range from mild to severe (Carroll et al., 2025; Holden et al., 2025). Dyslexia not only compromises educational outcomes but also adversely affects academic achievement and mental well-being. The cognitive and neural mechanisms underlying dyslexia remain debated. Cognitive accounts attribute dyslexia to deficits in phonological decoding, rapid automatized naming, or visuo-attentional skills (Goswami, 2015b; Valdois, 2022), whereas neurobiological theories implicate abnormalities in the magnocellular pathway (Qian & Bi, 2014), occipitotemporal or temporoparietal regions (Altarelli et al., 2013; Paulesu et al., 2001; Seki et al., 2001), or cerebellar dysfunction (Nicolson et al., 2001). Beyond these deficit hypotheses, dyslexia is now understood as a multifactorial, developmental, language-based difficulty shaped by the probabilistic interplay of genetic, cognitive–linguistic, neurobiological, and environmental influences over time (Lallier et al., 2017; Snowling & Hulme, 2025; Wolf et al., 2024).

Fluent reading involves the integration of orthographic, phonological, and semantic information to bridge written and spoken language (Huber et al., 2018). While reading processes share universal features across languages, script-specific variations arise due to differences in grapheme-phoneme mapping rules and orthographic complexity. Consequently, the neurobiological basis of dyslexia may vary cross-linguistically. English-speaking children with dyslexia typically exhibit atypical activation in temporo-occipital regions (Pugh et al., 2000; Temple, 2002), whereas Chinese individuals with dyslexia show abnormalities in the left middle frontal gyrus and right lingual gyrus (Siok et al., 2004, 2008). Cross-linguistic studies report both universal (Feng et al., 2020; Hu et al., 2010) and language-specific (Y. Li & Bi, 2022; Siok et al., 2008) neural markers of dyslexia.

A systematic synthesis focusing specifically on neural mechanisms underlying L2 reading impairment in children remains scarce. By addressing this gap, the present review provides timely insights into the neurocognitive mechanisms of L2 reading impairment. First, we outlined their behavioral manifestations and cognitive underpinnings. Next, we synthesized neuroimaging findings on children with L2 impairment and bilingual impairments, discussing potential deficit sources of L1–L2 commonality and divergence. Finally, we proposed future brain imaging research directions of L2 reading impairment. Investigating bilingual individuals is critical to disentangle shared versus distinct neural mechanisms between first and second language reading impairment, advancing understanding of the brain’s reading network and its relationship with reading impairment. While research on bilingual reading impairment spans various language pairs, the discussion of neural mechanisms in this review specifically centers on Chinese L1–English L2 learners. For two main reasons: first, the majority of current empirical studies have been conducted on this population; and second, the unique contrast between alphabetic and logographic orthographies provides a valuable lens for understanding both universal and language-specific neural mechanisms underlying reading impairment.

## 2. Behavioral and Cognitive Manifestations of L2 Reading Impairment

Substantial individual variability exists in L2 acquisition, with some learners exhibiting disproportionate reading impairment inconsistent with their cognitive capacity or motivation. A large-scale study of Chinese-English bilingual children in Beijing revealed dyslexia prevalence rates of 8.2% for L1 (Chinese) and 14.22% for L2 (English) (Y. Gao et al., 2019), highlighting the need to investigate L2-specific mechanisms. Notably, dissociations occur where impairment affects only one language (Wydell & Butterworth, 1999). These suggests that while certain aspects of reading impairment appear to be language-general in bilingual children, dissociations across different scripts likely stem from the unique cognitive demands imposed by each writing system (Perfetti et al., 2007). For Chinese–English bilinguals, the two languages represent fundamentally different writing systems. Chinese characters are organized within a square configuration, composed of strokes or radicals with high visual complexity and requiring more holistic processing, whereas English words follow a linear arrangement (X. Li et al., 2022b; Wu et al., 2012). Therefore, for individuals with Chinese as their L1 and English as their L2, L2-specific reading impairment may primarily be associated with the increased phonological demands of alphabetic language processing. It remains an open question whether similar dissociations would emerge in bilinguals whose L1 and L2 share the same orthographic type (e.g., two alphabetic or two logographic languages). Addressing this question could clarify whether these patterns are specific to Chinese–English bilinguals or reflect more general cross-linguistic phenomena.

Children with L2 reading impairment often show reduced fluency and accuracy, along with increased phonological and orthographic errors. For beginners, limited vocabulary is a key obstacle, and orthographic, phonological, and semantic processing all contribute to L2 reading development. According to connectionist models (Seidenberg, 2005), deficits in any of these components can lead to L2 reading impairment. Contributing factors include socioeconomic status, visual attention span (VAS), L1 characteristics (Fazio et al., 2021; Lallier et al., 2014; Sun et al., 2022), phonological awareness (Ho & Fong, 2005), and verbal working memory (Fazio et al., 2021). Prior studies have shown that phonological awareness influences reading accuracy in L2 and serves as a foundational skill that predicts the speed and efficiency of reading acquisition (Ho & Fong, 2005). In addition, verbal working memory has been found to impact L2 reading accuracy, with evidence indicating that children with L2 reading impairment exhibit poorer working memory performance compared to typically developing peers (Fazio et al., 2021). The VAS refers to the number of visual elements that can be simultaneously processed within a multi-element array. It has been shown to play a causal role in reading acquisition and is often impaired in individuals with dyslexia (Valdois, 2022). Evidence further suggests that learning an opaque L2 can enhance VAS skills, as observed in late Italian–English bilinguals (Venagli et al., 2025). Furthermore, L1 background modulates L2 processing. For instance, Arabic speakers struggle with word recognition, whereas Japanese speakers have difficulty with word integration when reading English (Fender, 2003), highlighting the possibility that L2 reading impairment may also be modulated by the characteristics of the learner’s L1.

Two theoretical frameworks dominate L2 reading impairment. The Linguistic Coding Differences Hypothesis (LCDH) posits shared difficulties in L2 and L1. Deficits in phonological, semantic, or syntactic processing in L1 can exert negative transfer effects on L2 acquisition, particularly in phonology (Sparks, 1995). Several studies have indicated that phonological awareness in L1 can transfer across languages and contribute to the development of L2 reading skills. For example, Shum et al. (2016) demonstrated that early L1 skills such as rapid automatized naming and rhyme awareness significantly predict L2 literacy outcomes. Other studies have also shown that L1 accuracy and speed in reading both real words and non-words can serve as reliable predictors of L2 reading impairment (Kormos et al., 2019). M. Li et al. (2022a) classified reading impairment into decoding and comprehension subtypes, showing that L1 difficulties in each area transferred to L2, which highlights the role of cross-linguistic transfer in shaping L2 reading development and underscores the importance of considering L1 reading profiles when evaluating L2 learning challenges.

In contrast, the Orthographic Depth Hypothesis and Psycholinguistic Grain Size Theory (PGST) emphasize script-specific demands: L2 impairment may stem from deeper orthography (Katz & Frost, 1992), mismatched grain sizes (Ziegler & Goswami, 2005), inadequate cognitive resources (e.g., working memory, Swanson et al., 2011), or insufficient exposure (Elbro et al., 2012). Crucially, whether “pure” L2 impairment reflects genuine impairment or environmental deprivation requires further scrutiny.

## 3. Neural Mechanisms of L2 Reading: Current Findings

Given that L1 and L2 reading share both universal and language-specific neural mechanisms, do L1 and L2 reading impairment similarly exhibit common and distinct neural substrates? Are the neural abnormalities in L2 reading impairment more consistent with those observed in the individual’s impaired L1 reading or with the neural patterns seen when the L2 is acquired as a native language?

Existing neuroimaging studies on L2 reading impairment have primarily focused on Chinese-English bilingual children. You et al. (2011) were among the first to investigate functional abnormalities in children with L2 reading impairment and found dyslexic children exhibited atypical activation in the temporoparietal and occipitotemporal regions. Specifically, reduced activation was observed in the left inferior occipital gyrus during orthographic processing and in the left angular gyrus during phonological processing compared to typically developing (TD) children (You et al., 2011). A subsequent study employing auditory stimuli found that children with L2 impairment showed weakened functional connectivity between the left superior temporal gyrus (STG) and left fusiform gyrus, though STG activities did not differ from TD (Meng et al., 2016). This suggests intact phonological representation but impaired phonological access in L2 reading impairment. Together, these findings indicate that the neural differences in L2-impaired learners resemble those in native English-speaking children with dyslexia (Meng et al., 2016; You et al., 2011). Further research using large-scale brain network analysis examined how functional networks organize and adapt during reading-related tasks in TD and L2-impaired children. During phonological tasks, impaired readers exhibited abnormally high local efficiency and modularity. Additionally, they demonstrated difficulties in network reconfiguration when switching between phonological tasks and resting states, evidenced by reduced changes in local efficiency and fewer module rearrangements (L. Liu et al., 2016).

Previous studies did not clarify the relationship between L1 and L2 reading impairment due to a lack of L1 ability assessment. Addressing this, H. Li et al. (2018a) categorized L2-impaired children into those with comorbid L1 impairment (PB group) and those with L2-specific impairment (PE group). The PB group showed gray matter abnormalities in the left fusiform gyrus, likely related to L1 impairment, while both groups exhibited anomalies in the left supramarginal gyrus (SMG), implicating it in L2 impairment independent of L1. Follow-up analyses in a TD cohort showed that SMG volume predicted L2 (but not L1) literacy, supporting its L2-specific role. Consistent with the PGST (Ziegler & Goswami, 2005), L2 impairment may stem from unmet language-specific demands. For instance, although English (L2) is more transparent than Chinese (L1), bilingual children often struggle with English grapheme-phoneme mapping, suggesting that differences in neural mechanisms are engaged when L2 employs a writing system distinct from those of the L1. Moreover, previous meta-analyses have shown that bilinguals are more likely to maximally recruit their L1 reading system during L2 reading when the two languages differ in orthographic systems (e.g., Chinese–English bilinguals), compared to when the two languages share greater orthographic similarity (e.g., two alphabetic languages) (H. Li et al., 2021).

## 4. Comorbidity Between L1 and L2 Reading Impairment

The existence of L2-specific reading impairment (with intact L1 abilities) suggests that L2 reading impairment may involve distinct mechanisms. Nevertheless, recent studies indicate that L1 reading impairment significantly increase the likelihood of L2 impairment. Specifically, children with L1 reading impairment exhibit a 36% comorbidity rate for L2 reading impairment (Y. Gao et al., 2019), with similar findings reporting a 40% co-occurrence rate (Chung et al., 2022). These results imply shared linguistic components between the two languages (Chinese L1 and English L2) despite their distinct writing systems.

Recent research by Cao et al. (2021), employing the “age- and reading-match” design, revealed that Chinese-English bilingual children with reading impairment exhibited common neural deficits across L1 and L2 (the left inferior temporal gyrus and the left precuneus), L1-specific deficits (in the left inferior parietal lobule), and L2-specific deficits (in the left ventral inferior frontal gyrus). Notably, dyslexic children showed significant differences between Chinese and English in the left ventral inferior frontal gyrus and left inferior parietal lobule, a pattern absent in TD children. This suggests that TD children effectively transfer L1 reading skills to L2, whereas dyslexics exhibit reduced cross-linguistic transfer, likely due to weaker foundational L1 proficiency. Behavioral data from the study corroborated this interpretation (Cao et al., 2021).

However, while this study examined bilinguals with reading impairment, it did not separately analyze L1-specific and L2-specific impairment subgroups. Zhang et al. (2025) has investigated whether L2 reading impairment possess unique neural mechanisms while accounting for L1 reading competence. The results showed that the difference of representational stability in the left middle temporal gyrus was specific to children with L2 reading impairment. However, the extent to which L1 and L2 reading impairment share universal versus language-specific neural markers warrants further exploration.

## 5. Universality and Specificity of L1 and L2 Reading Impairment: Underlying Mechanisms

Existing research on Chinese-speaking children suggests that L2 reading impairment are primarily related to phonological deficits independent of L1 reading abilities. These findings indicate distinct cognitive and neural substrates underlying L2 reading impairment. However, this account fails to explain cross-linguistic transfer effects. The phenomenon of reading impairment transferring from L1 to L2 implies that, in addition to phonological difficulties, other underlying difficulties may exist, which could potentially serve as the basis for cross-linguistic transfer. Such transfer effects reflect shared cognitive and neural mechanisms between L1 and L2. What factors contribute to the universality and specificity of reading impairment across languages? Reading, whether in L1 or L2, engages multiple cognitive processes, including not only phonological processing but also basic visual perception, eye movement control, and attention (Achal et al., 2016; Huber et al., 2018). Difficulties in any of these domains may lead to reading impairment. Current theoretical accounts of reading impairment can be categorized into four main perspectives.

First, the phonological deficit hypothesis posits that dyslexia stems primarily from impaired phonological processing (Black et al., 2017; Ramus & Ahissar, 2012; Ramus & Szenkovits, 2008; Xia et al., 2017). This domain-specific explanation suggests that deficient phonological awareness undermines reading ability (Danelli et al., 2013), with associated neural abnormalities observed in temporoparietal regions, including the posterior superior temporal gyrus, supramarginal gyrus, inferior parietal lobule, and angular gyrus (Linkersdörfer et al., 2012; Maisog et al., 2008; Richlan et al., 2013). The impact of phonological deficits may vary across languages, with greater relevance for languages heavily reliant on phonological processing. For example, research indicates that phonological awareness deficits are more strongly associated with reading impairment in English (an alphabetic language) than in Chinese (a logographic language) (Black et al., 2017; Xia et al., 2017). Supporting this, studies of Chinese-English bilingual children reveal that phonological awareness correlates significantly with English (L2) but not Chinese (L1) word reading (Ho & Fong, 2005), suggesting that phonological deficits may represent a language-specific cognitive basis for L2 reading impairment.

Second, the visual-attentional deficit hypothesis proposes that dyslexia arises from difficulties in visual-perceptual and attentional processes (Gori et al., 2014; Goswami, 2015a). These domain-general deficits manifest as abnormalities in visual search, visuospatial attention, and related functions that impact reading (Gori et al., 2016; Zhao et al., 2014). A specific instantiation of this account is the visual-attention span (VAS) deficit hypothesis, which argues that dyslexia reflects a reduced capacity to process multiple orthographic units simultaneously. Evidence reviewed by Valdois (2022) indicates that a limited VAS contributes causally to reading impairment across languages, independently of phonological skills. VAS performance relates to the activation of parietal regions within the dorsal attentional network, that these parietal regions are activated by VAS tasks. In addition, the neural correlates of visual-attentional deficits involve the magnocellular pathway, spanning the primary visual cortex to higher-order regions such as the inferior parietal lobule and left middle temporal gyrus (Qian & Bi, 2015; Qian et al., 2015). This framework explains why dyslexic children struggle with visually crowded text (Danelli et al., 2013) and likely represents a language-universal mechanism of reading impairment.

Third, the sensorimotor integration hypothesis attributes dyslexia to impaired motor-perceptual coordination, with proposed cerebellar involvement (Nicolson et al., 2001). This theory suggests that early motor learning deficits (e.g., in articulation or speech fluency) may disrupt subsequent phonological development and reading (Alvarez & Fiez, 2018; Ben-Yehudah & Fiez, 2008; Mariën et al., 2014; Nicolson et al., 2001). As a domain-general account, this perspective may explain cross-linguistic transfer effects in reading impairment. Supporting evidence comes from S. Li et al. (2018b), who found that bilingual individuals with reading impairment consistently exhibited rapid naming deficits in both languages, indicating shared cognitive mechanisms. Thus, L1 reading abilities may influence L2 development through common processing pathways (e.g., rapid naming), highlighting the interplay between universal and language-specific factors in reading impairment.

Finally, within the multifactorial framework, dyslexia is understood to arise from the combined influence and interaction of multiple risk factors, including phonological processing, rapid automatized naming (RAN), and oral language difficulties (Wolf et al., 2024). These risk factors, like dyslexia itself, are dimensional in nature and can vary in degree across individuals. Crucially, as emphasized by Catts and Petscher (2022), these complex interactions are further shaped by environmental influences such as instructional quality, linguistic context, parental support, and individual background. Within this perspective, the most prominent predictive factors include weaknesses in the phonological components of language and in processes indexed by naming speed, either independently or in combination. These vulnerabilities may further co-occur with additional factors, such as deficits in executive functioning and visual–orthographic processing.

In summary, each framework provides valuable but partial insights. The phonological deficit hypothesis highlights domain-specific linguistic impairments but may underrepresent visual and motor factors. The visual-attentional deficit hypothesis captures domain-general perceptual challenges but requires stronger integration with linguistic evidence. The sensorimotor integration hypothesis emphasizes motor-perceptual coordination but has less empirical support in L2 populations. According to the multifactorial hypotheses, dyslexia has been conceptualized within a dynamic and comprehensive framework. These perspectives suggest that our future understanding of reading impairment should not be confined solely to linguistic impairments but rather requires the consideration of multiple cognitive domains, such as visual attention span, executive control and automatization ability.

## 6. Methodological Advances and Future Directions

### 6.1. From Univariate to Multivariate and Network-Based Analyses

Most neuroimaging studies on L2 reading impairment to date have relied on traditional univariate methods, which examine brain regions or contrasts in isolation. However, differences in the neural processing of L1 and L2 may be reflected in the distinctiveness of neural activation patterns, rather than in the overall magnitude of regional activation (Weaverdyck et al., 2020). This underscores the utility of multivariate pattern analysis (MVPA), which is particularly sensitive to such fine-grained differences. Even in regions that are commonly engaged during both L1 and L2 processing, language-specific neural encoding may be present, as the pattern of neural responses to each language can differ within the same area. Employing MVPA therefore enables the detection of subtle differences in neural activation patterns associated with L1 and L2 processing.

Language represents a higher-order cognitive function, with growing evidence indicating that complex processes like reading emerge from the interaction and integration of multiple brain regions (Bassett & Gazzaniga, 2011; Park & Friston, 2013). Accordingly, a network-based approach is essential to understanding reading. Graph theory has been used to identify structural and functional network abnormalities in children with L1 reading impairment (Finn et al., 2014; González et al., 2016; Hosseini et al., 2013; K. Liu et al., 2015; Qi et al., 2016). Structurally, dyslexic children show higher local but lower global efficiency (K. Liu et al., 2015). Functionally, they exhibit increased local efficiency and clustering (Zhang et al., 2021), as well as abnormal connectivity within the visual pathway and between visual and attention-related regions (Finn et al., 2014). However, research on brain network abnormalities in L2 reading impairment remains limited

Future research should increasingly adopt MVPA and network-based connectivity models to assess how brain regions cooperate during reading in L2 impairment. These methods can reveal patterns of neural representation, and individual differences in brain activation patterns predictive of reading outcomes. Moreover, multivariate models allow for the integration of behavioral and neural data, offering a more holistic perspective on reading development and impairment.

### 6.2. Toward Naturalistic Reading Tasks

Most current studies employ simplified or highly controlled reading tasks (e.g., word recognition, lexical decision tasks) that may not generalize to naturalistic reading. To bridge the gap between laboratory and naturalistic reading, future work should extend beyond isolated word or sentence processing and examine naturalistic reading contexts, where comprehension depends on the coordination of multiple linguistic and cognitive subsystems (e.g., attention, working memory, executive control). These tasks can better reflect the demands of actual L2 literacy environments, which are more ecologically valid paradigms. Traditional General Linear Model (GLM) approaches present limitations in investigating the neural mechanisms underlying natural language processing. These methods fail to capture the continuous and dynamic nature of language processing, while conventional group analyses conflate individual characteristics, population-level features, and noise. In contrast, Inter-subject Correlation (ISC) analysis effectively reveals consistent neural responses across individuals during naturalistic stimulus processing (Hasson et al., 2012; Nastase et al., 2019). By aligning neural signals with input information consistently across participants, ISC identifies population-level neural representations of external stimuli.

### 6.3. Cross-Linguistic and Cross-Cultural Comparative Studies

Existing research indicates that English-speaking children with reading impairment primarily exhibit abnormalities in temporo-occipital regions (Pugh et al., 2000; Temple, 2002), whereas Chinese-speaking children with dyslexia show atypical activation in the left middle frontal gyrus and right lingual gyrus (Siok et al., 2004, 2008). Studies comparing English- and Chinese-speaking children with reading impairment have identified common neural deficits in the left middle frontal gyrus, left temporo-occipital region, and left angular gyrus (Hu et al., 2010). Furthermore, a direct cross-cultural comparison study has also observed commonalities between French- and Chinese-speaking children with reading impairment (Feng et al., 2020). Given the confounding effects of genetic and environmental factors in cross-cultural studies, bilingual populations represent the optimal sample for investigating the universality versus specificity of neural deficits in reading impairment.

L2 reading impairment is influenced by the structural characteristics of both L1 and L2, including orthographic depth, syntactic complexity, and semantic transparency. Future work should employ cross-linguistic and cross-cultural designs to compare how L2 reading impairment manifests across different L1-L2 pairings (e.g., alphabetic-to-logographic vs. alphabetic-to-alphabetic). Such studies can reveal differences and commonalities of neural signatures in reading impairment, and inform the development of culturally sensitive diagnostic tools and intervention strategies. Moreover, examining bilinguals with different proficiency profiles or literacy backgrounds can help clarify the role of L1 skills in shaping L2 reading outcomes.

### 6.4. Integration of Multimodal Neuroimaging Techniques

To deepen our understanding of the temporal and spatial dynamics of L2 reading, future studies should leverage the complementary strengths of different neuroimaging modalities. To date, functional near-infrared spectroscopy (fNIRS) studies have been conducted that provide high ecological validity and strong compatibility with electroencephalography (EEG), enabling the investigation of both shared and distinct neural correlates of first and second language processing (F. Gao et al., 2023). Combining functional magnetic resonance imaging (fMRI) with EEG or magnetoencephalography (MEG) can reveal both when and where neural processing breakdowns occur in L2 learners with impairments. For example, EEG or MEG can help identify delays in early visual word recognition or semantic integration processes. Moreover, multimodal neuroimaging is particularly well-suited to studying dynamic neural mechanisms during reading.

First, multimodal approaches can help track how different brain systems dynamically interact over time, and whether disruptions in their temporal coordination underlie the persistent challenges faced by children learning to read in a second language. Furthermore, integrating multimodal neuroimaging with computational modeling, such as neural decoding or network-based approaches, may provide mechanistic insights into how inefficient brain state transitions or atypical oscillatory patterns give rise to observable reading impairment. Finally, longitudinal designs that combine behavioral assessments with multimodal neural measures could reveal how developmental changes in neural dynamics support, or fail to support, the acquisition of fluent L2 reading, thereby informing targeted educational interventions and personalized remediation strategies.

### 6.5. Interventions for L2 Reading Impairment

An important avenue for future research concerns interventions for L2 reading impairment, encompassing both behavioral and neural approaches. Traditional behavioral programs (phonological awareness, grapheme–phoneme mapping, fluency, and morphology training) remain effective core treatments and should be adapted to target L2-specific demands (e.g., orthographic-to-phonological mapping in alphabetic L2s). Beyond phonology, previous work emphasizes the importance of visuospatial attentional skills (Valdois, 2022), suggesting that interventions targeting the visual attention span may provide additional benefits for L2 learners with reading impairment.

At the neural level, non-invasive brain stimulation (e.g., transcranial direct current stimulation, tDCS) has shown promise in modulating phonological processing and reading efficiency, particularly when applied over left temporoparietal regions implicated in assembled phonology (Xue et al., 2017). Recent studies further identify the left inferior parietal lobule as a potential neural substrate for L2 reading gains, suggesting that combined stimulation and L2-specific training targeting this region may be a fruitful direction (Zhang et al., 2023). Future research could also explore multimodal and integrative interventions that combine phonological, visuospatial, and sensorimotor training, potentially enhanced with neurofeedback techniques to dynamically tailor intervention to individual learners. Importantly, such approaches should consider developmental stage, L2 proficiency, and orthographic distance from the L1, paving the way for more personalized and effective interventions.

## Data Availability

No new data were created or analyzed in this study. Data sharing is not applicable to this article.
